# Plant Comparative and Functional Genomics

**DOI:** 10.1155/2015/924369

**Published:** 2015-12-31

**Authors:** Xiaohan Yang, Jim Leebens-Mack, Feng Chen, Yanbin Yin

**Affiliations:** ^1^Biosciences Division, Oak Ridge National Laboratory, Oak Ridge, TN 37831, USA; ^2^Department of Plant Biology, University of Georgia, Athens, GA 30602, USA; ^3^Department of Plant Sciences, University of Tennessee, Knoxville, TN 37996, USA; ^4^Department of Biological Sciences, Northern Illinois University, DeKalb, IL 60115, USA

Plants form the foundation for our global ecosystem and are essential for environmental and human health. With an increasing number of available plant genomes and tractable experimental systems, comparative and functional plant genomics research is greatly expanding our knowledge of the molecular basis of economically and nutritionally important traits in crop plants. Inferences drawn from comparative genomics are motivating experimental investigations of gene function and gene interactions. This special issue aims to highlight recent advances made in comparative and functional genomics research in plants. Nine original research articles in this special issue cover five important topics: (1) transcription factor gene families relevant to abiotic stress tolerance; (2) plant secondary metabolism; (3) transcriptome-based markers for quantitative trait locus; (4) epigenetic modifications in plant-microbe interactions; and (5) computational prediction of protein-protein interactions. The plant species studied in these articles include model species as well as nonmodel plant species of economic importance (e.g., food crops and medicinal plants).

*Evolution of Transcription Factor Gene Families Relevant to Abiotic Stress Tolerance*. The extant flowering plants have experienced multiple rounds of genome duplication and gene duplication is the primary source of gene family evolution. X.-L. Wang et al. in “Divergence of the bZIP Gene Family in Strawberry, Peach, and Apple Suggests Multiple Modes of Gene Evolution after Duplication” explored the evolutionary dynamics of the bZIP family, which contains transcription factors associated with plant tolerance to abiotic stress. They performed evolutionary analysis of the bZIP family in three rosaceous species in multiple aspects such as selection pressure on protein-coding sequences and genomic synteny. Another transcription factor gene family relevant to abiotic stress, the CCCH zinc finger family, was studied by W.-J. Chen et al. in “Significant Microsynteny with New Evolutionary Highlights Is Detected through Comparative Genomic Sequence Analysis of Maize CCCH IX Gene Subfamily.” They performed comparative analysis of the CCCH IX subfamily in three cereal grain species (i.e.,* Zea mays*,* Oryza sativa*, and* Sorghum bicolor*) and found that segmental duplication has played an important role in the expansion of this gene family. Their analysis also indicates that deletions, multiplications, inversions, and purifying selection have contributed to the evolution of the CCCH IX subfamily.

*Plant Secondary Metabolism*. Plants produce a wide range of secondary metabolites that underpin functional diversity in plants. Gene expression profiling through transcriptome sequencing is a powerful approach for understanding the molecular basis of plant secondary metabolism. H. Tian et al. in “Analysis of* Polygala tenuifolia* Transcriptome and Description of Secondary Metabolite Biosynthetic Pathways by Illumina Sequencing” analyzed expression of secondary metabolite biosynthetic genes in* P. tenuifolia*, a well-known medicinal plant, using RNA-seq approach. Their analysis revealed candidate genes that are potentially involved in biosynthesis of several important secondary metabolites such as triterpene saponins and phenylpropanoid. Similarly, R. Li et al. in “*De Novo* Transcriptome Sequencing of the Orange-Fleshed Sweet Potato and Analysis of Differentially Expressed Genes Related to Carotenoid Biosynthesis” performed RNA-seq analysis of secondary metabolism in* Ipomoea batatas*, an important food crop. Through comparing the global gene expression profile in relation to the differences in the carotenoid content of two* I. batatas* cultivars, they identified more than 50 genes potentially involved in carotenoid biosynthesis. Also, Y. Wei et al. in “Genome-Wide Identification of Genes Probably Relevant to the Uniqueness of Tea Plant (*Camellia sinensis*) and Its Cultivars” performed comparative analysis of RNA-seq data in several species of the genus* Camellia*, an important source for tea production. They identified differentially expressed genes relevant to the biosynthesis of flavonoid, theanine, and caffeine. Furthermore, their sequence comparison revealed nonsynonymous mutations that are potentially related to the diversity between the two cultivars of* C. sinensis*.

*Transcriptome-Based Markers for Quantitative Trait Locus*. Quantitative trait locus (QTL) analysis has been widely used for elucidating the genetic basis of complex traits in plants. Molecular makers are prerequisites for QTL analysis. QTL makers can be developed from either genome sequences or transcriptome sequences. Development of genome-based markers requires genome-sequencing data, which are available only in model plant species or major crop species. For nonmodel plant (crop) systems, transcriptome-based markers can be a better choice for QTL analysis with a limited budget. E. S. Seong et al. in “Expressed Sequence Tags Analysis and Design of Simple Sequence Repeats Markers from a Full-Length cDNA Library in* Perilla frutescens* (L.)” developed simple sequence repeats (SSR) markers based on approximately 1,000 expressed sequence tags (ESTs) derived from cDNA libraries for this member of the mint family used in traditional Asian medicine. They identified 18 SSR makers that could be very useful for understanding of genomic basis of medicinal function in* P. frutescens*. Recent advance in next-generation sequencing technology greatly enhances the capability for molecular marker development. Q. Ding et al. in “Characterization and Development of EST-SSRs by Deep Transcriptome Sequencing in Chinese Cabbage (*Brassica rapa* L. ssp.* pekinensis*)” identified 10,420 SSR markers from 51,694 nonredundant unigenes assembled from RNA-seq data. This large set of SSR makers could facilitate genome-wide discovery of QTLs in Chinese cabbage. Also, R. Li et al. in “*De Novo* Transcriptome Sequencing of the Orange-Fleshed Sweet Potato and Analysis of Differentially Expressed Genes Related to Carotenoid Biosynthesis” identified 1,725 SSR markers in the transcriptome data for sweet potato.

*Epigenetic Modifications in Plant-Microbe Interactions*. While it is widely accepted that genetics governs plant growth, development, and response to environment, an increasing number of studies have showed that epigenetics also plays an important regulatory role in plants. The paper by K. Melmaiee et al. entitled “Quantification and Gene Expression Analysis of Histone Deacetylases in Common Bean during Rust Fungal Inoculation” revealed that epigenetic modification via histone deacetylases is involved in the response of common bean to rust fungal inoculation. The results from this paper provide new insight into the molecular mechanism underlying plant-microbe interactions.

*Computational Prediction of Protein-Protein Interactions*. Protein-protein interaction (PPI) is an important molecular mechanism underlying various biological processes. Computational prediction of protein-protein interactions based on protein sequences is a straightforward approach to the utilization of whole-genome gene annotation for the global view of protein-protein interaction network in an organism. Various algorithms have been developed for protein sequence-based PPI prediction, though with limited success. J. Yao et al. in “PPCM: Combing Multiple Classifiers to Improve Protein-Protein Interaction Prediction” present a machine learning approach for PPI prediction based on various features derived from protein sequences. Their results demonstrated that integration of multiple features could significantly improve the PPI prediction accuracy as compared with prediction classifiers based on individual features. This novel approach has a great potential for PPI prediction in nonmodel organisms, including plant species.

